# Less is more: Low-strength type I interferon signaling enhances CAR T cell therapy

**DOI:** 10.1016/j.omton.2026.201351

**Published:** 2026-10-13

**Authors:** Erting Tang, Jun Huang

**Affiliations:** 1 Pritzker School of Molecular Engineering, University of Chicago, Chicago, IL 60637, USA; 2 Committee on Immunology, University of Chicago, Chicago, IL 60637, USA; 3 Committee on Cancer Biology, University of Chicago, Chicago, IL 60637, USA

## Main text

Current CAR T cell therapies produce durable remissions in B cell malignancies, yet primary resistance and relapse remain major challenges.^1^^,^^2^ Most efforts to improve efficacy have focused on the receptor or the genome encoding it, through new receptor and costimulatory designs, additional genetic modifications, rewiring of intracellular signaling, phenotype-directed differentiation, or combination regimens.^3^ These strategies are powerful, but most introduce a new genetic element or a novel biologic agent and with it, a new engineering, manufacturing, and regulatory burden. In our recently published study, “Low-Strength Type I Interferon Signaling Promotes CAR T cell Treatment Efficacy,” in *Cancer Immunology Research*,^4^ we took a different approach: rather than further redesigning the CAR, we asked whether the functional state of an already FDA-approved CAR T cell product could be improved during manufacturing by a brief cytokine intervention. The pathway was chosen for us by the clinical data rather than by design: type I interferon (IFN-I) signaling emerged from the infusion products themselves, and IFN-I itself is FDA-approved, with decades of clinical use in malignancies, autoimmune disease, and viral hepatitis. Pairing an approved cell product with an approved cytokine and leaving CAR architecture untouched, this strategy may simplify clinical translation and integrate readily into existing manufacturing workflows.

The key clinical observation was direct. Using single-cell transcriptomics, we profiled the axicabtagene ciloleucel infusion products of eight patients with relapsed or refractory diffuse large B cell lymphoma and found that products from complete responders carried consistently stronger IFN-I response programs than those from non-complete responders (Figure 1A).^4^ The same enrichment held in an independent published cohort of 15 patients. Across both cohorts, the signature was evident in individual patients and in nearly every T cell subset we resolved. This informed us that IFN-I signaling is not merely a correlative signature but a candidate pathway we could deliberately tune during manufacturing.

**Figure 1:**
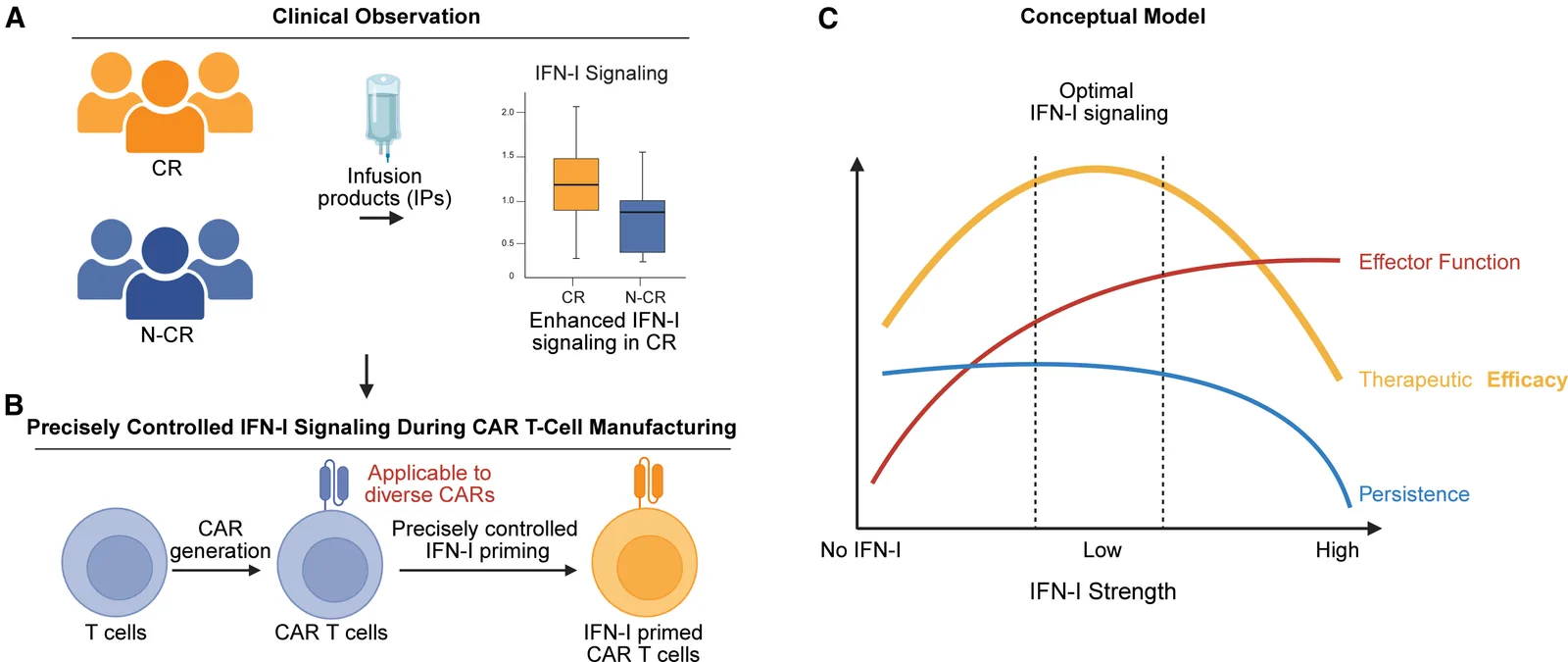
A conceptual framework for strength-dependent IFN-I signaling during CAR T cell manufacturing (A) Single-cell transcriptomic analysis of CAR T cell infusion products revealed enhanced type I interferon (IFN-I) response programs in complete responders (CRs) compared with non-complete responders (N-CRs). (B) Guided by this clinical observation, we introduced a precisely controlled IFN-I priming step during *ex vivo* CAR T cell manufacturing. This strategy is broadly applicable across diverse CAR designs. (C) Proposed conceptual model illustrating the strength-dependent effects of IFN-I signaling on CAR T cell function. Increasing IFN-I signaling progressively enhances effector function but also reduces CAR T cell persistence, resulting in an optimal signaling window that balances these opposing effects and maximizes overall therapeutic efficacy.

This raised a practical question: could short-term IFN-I exposure during manufacturing yield more effective CAR T cells (Figure 1B)?

IFN-I cytokines are best known for their antiviral and immunomodulatory activity, and they shape T cell responses directly through the type I interferon receptor (IFNAR). Engagement of IFNAR activates JAK-STAT signaling and promotes T cell activation, effector differentiation, and antitumor function. In CAR T cells, however, the published effects are strikingly inconsistent: some studies report improved cytotoxicity and tumor control,^5^ whereas others report apoptosis, dysfunction, and loss of persistence.^6^^,^^7^^,^^8^ We reasoned that these results might not be contradictory and that what mattered was not whether the pathway was engaged but how strongly.

Our data show that the effects of IFN-I on CAR T cells depend on signaling strength. More IFN-I did not simply produce better CAR T cells. Instead, IFN-I imposed a trade-off between enhanced cytotoxicity and reduced viability. A brief, five-day exposure to IFN-α2 during expansion, followed by complete washout before harvest, enhanced CAR T cell cytotoxicity at low strength while preserving viability, whereas high strength drove cytotoxicity higher at the cost of viability and, ultimately, persistence. The infused product therefore carries no residual interferon, avoiding the systemic toxicities that have long constrained interferon therapy. These findings suggest that optimizing, rather than simply increasing, the stimulation strength is what maximizes CAR T cell efficacy and support a model in which tuning IFN-I signaling to an intermediate window balances effector function against persistence (Figure 1C).

Importantly, this principle proved robust across different CAR constructs, cancer types, and donor sources. The benefits of low-strength IFN-I priming were consistently observed in CAR T cells bearing either CD28 or 4-1BB costimulatory domains, and so, they were not restricted to a particular CAR design. Low-strength priming prolonged survival and improved tumor control in xenograft models of both diffuse large B cell lymphoma and B cell acute lymphoblastic leukemia. CAR T cells generated from a patient leukapheresis product showed the same functional gains as those from healthy donors. The benefit was also durable: when mice were rechallenged with tumor after initial disease control, low-strength-primed CAR T cells sustained better tumor control and were present at higher numbers in blood and spleen.

This drop-in strategy is simple, effective, and adaptable. Because it modifies T cell state rather than CAR architecture, low-strength IFN-I priming can, in principle, be applied across diverse CAR designs and manufacturing workflows without any other change. It therefore complements, rather than replaces, ongoing advances in CAR engineering and in cytokine conditioning.

Our work also shows how clinical single-cell data can guide cell engineering: a response-associated transcriptional program in patient infusion products was converted into a testable manufacturing intervention and then validated functionally. This bedside-to-bench-to-bedside workflow may help identify other tunable pathways that improve CAR T cell efficacy.

As strategies for improving CAR T cell therapy grow increasingly sophisticated, our findings argue for a straightforward principle: during manufacturing, immune signaling should be optimized rather than maximized. Calibrating a well-characterized, clinically approved cytokine to its optimal signaling window may therefore represent a practical and readily translatable route to more effective and more durable cell therapies.

## Declaration of interests

E.T. and J.H. are listed as inventors on a pending US provisional patent application (63/789,043) covering interferon-enhanced CAR T cells. E.T. and J.H. report grant support from the NIH and the American Cancer Society during the conduct of the study.
